# Think global, act local: the experience of Global Fund and PEPFAR joint cascade assessments to harmonize and strengthen key population HIV programmes in eight countries

**DOI:** 10.1002/jia2.25125

**Published:** 2018-07-22

**Authors:** Tiffany A. Lillie, James Baer, Darrin Adams, Jinkou Zhao, R. Cameron Wolf

**Affiliations:** ^1^ FHI 360/LINKAGES Washington DC USA; ^2^ Independent consultant FHI 360/LINKAGES London UK; ^3^ Johns Hopkins Bloomberg School of Public Health Baltimore MD USA; ^4^ The Global Fund to fight AIDS, Tuberculosis and Malaria Geneva Switzerland; ^5^ U.S. Agency for International Development Washington DC USA

**Keywords:** HIV, key populations, assessment, donors, collaboration

## Abstract

**Introduction:**

The Global Fund and the US President's Emergency Plan for AIDS Relief (PEPFAR) are major donors to HIV services with key populations (KPs) to achieve the UNAIDS 95‐95‐95 epidemic control goals. The programmes they fund are not always well aligned or coordinated, decreasing their effectiveness. Joint assessments are designed and led by LINKAGES, a project funded by PEPFAR and the US Agency for International Development, to improve coordination among donors and on‐the‐ground implementation of KP HIV programmes. Joint assessments help identify barriers that prevent KPs from accessing interventions along the cascade of prevention, diagnosis and treatment services, and provide recommendations to improve and align programmes. Detailed reports from eight assessments in Malawi, Cameroon, Swaziland, Haiti, Angola, Nepal, Côte d'Ivoire and Botswana were analysed for thematic challenges, and recommendations are presented. The purpose of the paper is to identify commonalities across KP HIV programmes that were found through the assessments so others can learn and then strengthen their programmes to become more effective.

**Discussion:**

The joint cascade assessments offered countries feedback on HIV programme challenges and recommendations for strengthening them at national, subnational and local levels. Shared intervention areas included: (1) robust population size estimates to inform service delivery targets and to budget resources for KP outreach; (2) accessible and KP‐friendly services most relevant to individuals to increase retention in the HIV cascade; (3) decentralized, community‐based services for HIV testing and antiretroviral therapy, and new approaches including self‐testing and PrEP; (4) addressing structural issues of stigma, discrimination and violence against KPs to create a more enabling environment; and (5) more effective and continual tracking of KPs across the cascade, and coordinated, harmonized monitoring tools and reporting systems between donor‐funded and national programmes.

**Conclusions:**

The assessment teams and country stakeholders viewed the assessments as a best practice for coordinating donor‐funded programmes that may overlap or inefficiently serve KPs. Global and national HIV programmes need investments of time, resources, and commitment from stakeholders to continually course‐correct to align and improve programmes for sustained impact. The type of continued partnership demonstrated by the joint assessments is key to address HIV among KPs globally.

## 1 Introduction

In all countries affected by the HIV epidemic, key populations (KPs) – including sex workers, men who have sex with men (MSM), people who inject drugs, transgender people, and people in prisons and other closed settings – bear a disproportionate burden of HIV infection 1, 2, 3, 4, 5 and face formidable barriers to accessing services for HIV prevention, diagnosis, treatment and care. These barriers include stigma and discrimination, restrictive laws, violence and human‐rights violations 6. Many KP HIV programmes have insufficient scale, scope and quality to overcome these barriers. KPs are a high priority in efforts to prevent new HIV infections and achieve the ambitious UNAIDS 95‐95‐95 treatment goals by 2030 7. The Global Fund to Fight AIDS, Tuberculosis and Malaria (GF) and the US President's Emergency Plan for AIDS Relief (PEPFAR) are working to improve KP programmes based on World Health Organization (WHO) general and KP‐specific guidelines and are the largest funders of KP HIV services in many low‐ and middle‐income countries 8, 9, 10, 11, 12, 13, 14.

To help improve in‐country implementation responses, GF and PEPFAR have conducted joint KP HIV cascade assessments with teams of international donors and implementers, national governments and ministries of health, local implementing partners and representatives from KP groups. These assessments focus on aligning geographical areas, technical approaches, service packages, referral networks, targets and monitoring systems. Joint assessments can help address many of the complex challenges that arise during implementation and coordination of multiple KP programmes within a country. They also provide an opportunity to address on‐the‐ground challenges and advocate for delivery of HIV services to KPs at scale.

The joint assessments are designed and led by *Linkages across the Continuum of HIV Services for Key Populations Affected by HIV* (LINKAGES), a project funded by PEPFAR and the US Agency for International Development (USAID). LINKAGES has worked in over 30 countries to accelerate the ability of partner governments, civil society organizations, and private sector healthcare service providers to plan, deliver and optimize a package of comprehensive services, at scale, for HIV prevention, diagnosis, treatment and viral suppression for KPs. In most of these countries, GF also has active grants for KP HIV programmes.

LINKAGES has adopted the “cascade” of HIV services, a framework to track the movement of individuals across the continuum of care provided by community and health services. Country programmes supported by LINKAGES are monitoring their programmes using the cascade, based on WHO consolidated guidance 8. The framework views service delivery as going beyond traditional HIV services to include crosscutting topics such as sexual and reproductive health and critical enablers (e.g. laws, policies, community empowerment, and violence prevention and response) 8, 9, 10, 11, 12, 13, 14.

### 1.1 Implementing joint cascade assessments for key populations

The broad goals of joint KP cascade assessments are to: (1) determine the extent to which the services cascade is working for KPs by understanding the service flow and strategies within a project area, including services delivered by nongovernmental organizations (NGOs), community‐based organizations (CBOs) and clinics, government sites and other facilities, and hybrid models; (2) identify and analyse the causes of “leaks” in the system, where KPs are lost to follow‐up or are unable to access essential commodities and services, and recommend solutions that can be replicated and scaled up; and (3) identify possibilities for linking and aligning the monitoring systems of different providers at unique stages of the cascade.

This joint cascade assessment method is not intended to be a research activity generalizable across a country, nor a data audit or data validation tool. It is a rapid, stocktaking exercise using a convenience sample of KP HIV programmes within the country that feeds practical recommendations directly back into programmes and decision makers for immediate action and quality improvement. Community and clinical sites (i.e. both donor‐supported and public health facilities) are selected based on where US Government agencies implementing PEPFAR (i.e. the US Centers for Disease Control and Prevention and USAID) and GF are funding KP programmes, logistical ease and time constraints. Splitting the assessment team into smaller sub‐teams allows for multiple geographic locations and sites to be visited and reviewed at the same time. The assessment provides a solid basis for local implementers and national‐level programmes to advocate for the adoption of best practices and innovations with government and donors. Assessments are a consensus‐building process among multiple stakeholders, whereby they may agree on common approaches to harmonize technical and monitoring and evaluation (M&E) strategies, leading to programmatic and budget efficiencies.

Between February 2016 and September 2017, eight joint cascade assessments were conducted in Malawi, Cameroon, Swaziland, Haiti, Angola, Nepal, Côte d'Ivoire and Botswana. For this commentary, we reviewed and analysed all out‐brief presentations and full reports to identify commonalities observed in at least three countries. Common themes in challenges and recommendations at programme, national and donor levels, at each step in the cascade of services are highlighted. Findings were shared with representatives from in‐country implementation teams for validation. While the presentations focused on strengths as well as challenges, here we present and discuss the challenges and recommendations that were common to at least three countries.

The purpose of the paper is to identify commonalities across several KP HIV programmes that were found through the assessments so others can learn and then strengthen their programmes to become more effective.

## 2 Discussion

The KP HIV cascade assessments were the first formal exercises that built cross‐cooperation for KP programming among these major donors and stakeholders in the countries where they were conducted. The assessments were meant to develop a shared vision through a collaborative and participatory appraisal of the national KP programme, as implemented through local partners.

The assessment teams categorized challenges and recommendations using the HIV cascade framework: reach, HIV testing services, linkages to treatment, treatment, retention and viral suppression, monitoring and crosscutting issues (Table 1). The findings are summarized below.

**Table 1:** Primary challenges identified, and recommendations of joint cascade assessment teams

Stage of cascade	Challenges	Recommendations – programme level	Recommendations – national level	Recommendations – donor level
Identify KPs	Unreliable KP size estimates (S, A, CI, B) Targets set by donors not based on local‐level mapping and size estimation (S, H, CI, B)	Validate hot spot mapping (S, H, A) Regular revalidation of KP numbers at hot spots (N, CI, B)	Develop a system to determine national‐level estimates for KPs (M, A, CI, B)	Develop a system to determine national‐level estimates for KPs (M, A, CI, B)
Reach KPs	Insufficient number of peer outreach workers (i.e. KP individuals trained to do outreach) (Ca, A, N, CI, B) Lack of strategy for outreach to KP individuals who do not frequent physical hot spots (A, CI, B) Stock outs of condoms, limited availability of some commodities, e.g. female condoms, lubricants (S, H, A, N, CI, B)	Expand number of peer outreach workers (Ca, A, CI, B) Improve supervision and introduce microplanning (i.e. outreach and support plans based on the mapping/size estimation, and individual KP members’ risk assessment and needs) (Ca, S, H, A, N, CI, B) Systematize risk assessments and follow‐up, to identify harder‐to‐reach KPs (H, N, CI, B) Develop online outreach strategy (Ca, A, CI, B) Improve understanding among KPs of post‐exposure prophylaxis, treatment literary, viral load (“undetectable = untransmittable”), and violence reporting and response during outreach sessions (Ca, S, H, A, N, CI, B)	Establish or apply national standard for ratio of peer outreach workers to KPs (Ca, H, CI, B) Update national package of services to standardize and align with WHO KP guidelines, including PrEP (S, H, A, CI, B) Expand and integrate KP issues into national STI guidelines based on WHO guidelines (Ca, A, B) Ensure adequate stock of STI drugs, condoms/ lubricants, STI & HIV test kits, reagents and ARVs (Ca, S, A, N, CI, B)	Streamline and harmonize outreach package to include targets, geographical coverage areas, curricula, educational materials, peer outreach worker scope of work and incentives, and the package of services for KPs (M, Ca, S, H, N, CI, B)
KPs know status (test/retest)	Difficulty in accessing testing (Ca, N, B) Low HIV case‐finding rates (A, N, CI) Referrals to public facilities not always completed (Ca, A, CI, B)	Promote testing through improved messaging in all client communications (Ca, A, N) Provide multiple avenues for testing (e.g. community‐based, lay provider testing) (M, Ca, S, H, N, CI, B) Explore new approaches (self‐testing, lay‐administered oral fluid/finger‐prick tests) (Ca, S, A, N, CI, B) Integrate HIV testing with STI screening (Ca, H, A, B) Encourage partner testing for KP individuals living with HIV (A, N, CI, B) Use microplanning to regularize contact with KP individuals and remind them to be tested. Conduct individualized risk assessments to refine messaging based on KP individuals’ needs (Ca, S, H, N, CI, B) Offer PrEP (Ca, CI, B)	Ensure multiple avenues for testing are available (mobile, fixed sites, community‐based, lay provider testing) (M, Ca, S, H, N, CI, B) Explore new approaches (self‐testing, lay‐administered oral fluid/finger‐prick tests) (Ca, S, N, CI, B)	
Enrol KPs living with HIV in care, initiate ART	Complexity, time and expense of enrolment procedures (Ca, H, A, N) KP individuals’ denial about HIV diagnosis (A, N, CI, B) Insufficient number of peer navigators (i.e. trained KP individuals living with HIV who provide support for ART enrolment and adherence) (Ca, S, A)	Offer peer navigators to accompany KPs for ART enrolment, boost treatment literacy and support ART adherence (M, Ca, S, H, A, N, CI, B) Institute or strengthen “test and treat” to speed up initiation of ART (M, Ca, S, A, N, CI, B) Offer community‐based ART (M, Ca, S, H, A, N, CI, B) Offer psychosocial support (counselling) for the newly diagnosed (Ca, A, B) Formalize relationship between KP programmes and HIV clinics (Ca, S, A)	Increase and diversify the number of ART sites to make it easier for KP individuals living with HIV to initiate and stay on treatment (Ca, S, N, CI) Dispense multi‐month antiretroviral (ARV) prescriptions and ensure supply for stable clients (A, N, CI, B)	Provide additional technical assistance and guidance documents on “test and treat” to providers (M, Ca, S, H, A, N, CI, B)
Sustain on ART, suppress viral load	Need for monthly visits to fill ARV prescription (Ca, A, CI) Need for nutritional support (Ca, H, A, N) Mobility of KPs increases loss to follow‐up (S, N, CI, B) Delay in receiving viral load testing results; testing not always done reliably or recorded in patient records (H, N, CI) Lack of KP understanding that “undetectable= untransmittable” (S, A, N, CI, B)	Offer three‐month ARV prescriptions (Ca, A, N, CI, B) Reinforce treatment literacy programmes (Ca, S, H, A, N, CI, B) Establish systems to send appointment reminders, follow up on missed appointments (Ca, N, B) Decentralize viral load testing (H, N, CI) Offer nutritional support for those starting ART (Ca, S, H, N, A)	Explore use of GenXpert machines for viral load testing at point of service (A, N, CI, B)	
Programme monitoring and data use	Lack of coordination between implementing partners and between donors in data collection tools, indicators, and programme databases (Ca, S, H, A, N, CI, B) Duplication of KP individuals when recording services delivered (Ca, H, A, N, CI, B) Data not secure, confidential, or backed up (Ca, N, B) Data reported up without analysis for programme improvement at local level (M, N, CI, B)	Introduce/strengthen UICs (M, Ca, S, H, A, N, CI, B) Design coordinated computerized management information systems (H, N, B) Develop protocols for data security within computerized systems (passwords, back‐ups) and in paper copies (locked filing cabinets), and code of confidentiality for staff, including KP individuals involved in outreach (Ca, H, A, N, B) Provide training on data analysis and use for real‐time decision making and programme improvement (M, Ca, A)	Harmonize data collection forms and indicators at national level (M, H, A, CI)	Improve coordination and alignment between implementing partners, public health facilities and donors, in data collection tools, tracking systems (UICs), indicators and programme databases (Ca, S, H, A, N, CI, B) Support development of M&E systems at programme level with harmonized indicators; UICs across cascade of services; electronic systems; ensure programme‐level systems are compatible with national M&E system (M, Ca, S, H, A, N, CI, B) Create communities of practice by increasing technical exchanges between implementing partners through a national‐level technical working group or other fora (e.g. GF Country Coordinating Mechanism) (M, A, CI)
Stigma, discrimination, and violence	Widespread stigma, discrimination by multiple power holders (M, Ca, S, H, A, N, CI, B) Frequent violence (M, Ca, S, A, N, B) Discrimination within KP communities (M, A, CI, B) Poor reporting and follow‐up to violence (A, CI, B) Limited access to PEP (Ca, H, A, CI, B)	Training and sensitization, especially of police and health care providers (M, Ca, S, H, A, N, CI, B) Increase knowledge and use of PEP (Ca, H, A, CI, B) Training of KPs on their legal and civil rights (H, A, N, CI, B) Systems to track and report stigma, discrimination, and violence (M, Ca, A, N, CI) Community‐based violence response systems (M, S, H, A, N)		
Community empowerment and engagement	Low motivation/high turnover of peer outreach workers (H, N, CI, B)	Establish/expand drop‐in centres (H, CI, B) Train peer outreach workers and navigators, develop systems for peer progression and recognition (H, N, CI, B)		Align incentives for peer outreach workers between programmes (H, CI, B)

M, Malawi; Ca, Cameroon; S, Swaziland; H, Haiti; A, Angola; N, Nepal; CI, Côte d'Ivoire; B, Botswana.

### 2.1 Reaching KPs

Having strategies for outreach to KPs that are founded on trust and confidentiality is an important first step for HIV programmes. LINKAGES recommends a peer outreach worker (POW) to peer contact ratio of 1:50 for female sex workers (FSW) and 1:30 for MSM to provide frequent, high‐quality, individualized services based on international guidance 9, 10, 11, 12, 15, 16. In most assessments, it was found that there were too few POWs to adequately cover KPs in known hot spots, leading to irregular or poor levels of contact with KPs. Microplanning is based on the results of the hot spot mapping and size estimations, which provide a basis for planning and implementation, such as where drop‐in centres and testing sites should be located, how many POWs are needed, and where/how to focus resources and services based on population numbers 15, 16. Microplanning emphasizes individualized risk assessments to better target and provide services based on need. Another challenge identified was the lack of formal strategies for contacting harder‐to‐reach sub‐populations, such as MSM who do not gather at physical hot spots, but instead go online or use virtual networks.

Recommendations to address these challenges included recruiting and training more POWs to improve outreach ratios. Improving microplanning was recommended in most countries to ensure that KPs were covered by a standardized package of services that also addressed their individual needs. Offering post‐ and pre‐exposure prophylaxis (PEP and PrEP) was also recommended for inclusion in the standard package of services, as these services have been shown to increase demand for services 10, 11, 17, 18. Programmes were encouraged to develop strategies for hard‐to‐reach populations, including Internet/virtual outreach, and enhanced peer outreach approaches that incentivize recruitment through social networks, which have been found to increase demand for HIV testing and other services 19, 20, 21, 22.

### 2.2 KPs know their HIV status

There were three major challenges in reaching the first 95 goal: (1) lack of accessible KP‐friendly testing services; (2) lower case‐finding rates than expected targets (i.e. low rates of positive HIV test results) because of saturation within existing networks and difficulties identifying and engaging KP individuals at highest risk; and (3) HIV testing referrals made in the community frequently not being completed or properly tracked.

A common recommendation was to increase HIV testing modalities to make them more accessible by expanding community‐based testing, training more lay workers to conduct testing and increasing the number of testing options through drop‐in centres and mobile clinics 23, 24. The use of new approaches, such as lay‐provider HIV testing using finger‐prick or oral‐fluid tests or making self‐test kits available to KPs, was recommended based on global guidance 25, 26. If community‐based or self‐testing were not available, it was recommended to train and support POWs and peer navigators (i.e. trained KP individuals, themselves often living with HIV (KPLHIV), who provide support for antiretroviral therapy (ART) adherence) to offer accompanied referrals from community programs to health facilities to ensure services are linked 23, 24, 27. To address low case finding, strategies such as enhanced (network‐ and performance‐based) peer outreach and using data to better target those at higher risk were proposed to reach and test KP individuals who had not been previously been identified 28, 29, 30, 31. Programmes also were encouraged to increase index partner testing of KPLHIV of sexual and injection contacts to improve HIV case finding 28, 32, 33.

### 2.3 KPs living with HIV are enrolled in treatment

All countries reported having too few trained KP individuals to support referrals and linkages from testing to treatment, especially by accompanying KPLHIV to the treatment facility. Significant barriers to treatment initiation found in the assessments and literature included stigma and discrimination, internalized stigma, poor nutrition and substance abuse, along with lack of understanding of the full benefits of treatment, not only for the KPLHIV, but also as prevention for their partners 34, 35, 36. Another challenge commonly identified was the time to initiate ART after being newly diagnosed. The inconvenience and cost of travel to ART clinics and the high fees sometimes charged for appointments and tests were related barriers.

To improve ART initiation rates, the assessment teams and global experts recommended that programs provide community‐based support for ART initiation 23, 37. Further recommendations were for accompanied referrals to ART clinics whose staff have been formally sensitized and trained to work with KPLHIV, and support groups for KPLHIV. Treatment literacy education by POWs and those who provide testing was also stressed, so that KP individuals would understand the importance of treatment should they test positive and be supportive of their peers living with HIV 38. All assessments and global guidance recommended implementing or scaling up “test and treat” programmes that offer *immediate* ART initiation, and decentralizing distribution of antiretroviral drugs (ARVs) to stable patients, for example dispensing to stable patients by community‐based POWs or peer navigators and at drop‐in centres 37.

### 2.4 KP individuals living with HIV are sustained on ART and have suppressed viral load

The limited dosages of ARVs dispensed, typically enough for only one month, even for stable patients, were a serious challenge to retention. Inflexible appointment schedules and long waiting times at clinics made it hard for KP individuals to adhere to monthly appointments. Sometimes multi‐month prescriptions were provided, but limited supply meant that pharmacies would dispense only one or two months’ worth of antiretrovirals (ARVs) Nutritional support was also noted as a challenge, especially for those initiating ART who needed access to regular meals to help them tolerate the new drugs. In some sites, laboratory results on viral load could take up to a month to be communicated to providers, who sometimes did not use them for clinical monitoring. KP individuals in most countries did not understand that a sustained undetectable viral‐load status meant that they would have virtually no risk of transmitting HIV to a sexual partner.

Recommendations to counter these challenges included community‐based ART distribution following WHO's differentiated models of care 37 through various channels, and three‐month ART prescriptions for KP individuals who were stable in their treatment. In almost all the assessments, it was recommended that treatment literacy programmes help clients understand the importance of a suppressed viral load and thus adherence to their medication 38 through new communication messages and campaigns tailored for this purpose. Appointment reminders via text messaging and systems to track missed appointments were also recommended.

### 2.5 Programme monitoring and data use

It was a common challenge that programme targets were set using dated or unreliable KP size estimations, which resulted in the perception that programmes were either under‐ or overachieving based on poor denominators. It was recommended that some programmes conduct and validate mapping and size estimation to plan implementation in their assigned geographical areas 15, 16. A concurrent, robust mechanism to determine national‐ and sub‐national level KP estimates are necessary to accurately plan and implement programmes, streamline activities and reduce service duplication. Countries should follow WHO guidelines to estimate KP population size 20, 39.

A significant challenge for data collection and monitoring included the use of differing forms and systems across implementing partners, and a lack of alignment between the data required for reporting on programmes to the government (e.g. for clinical services) and those needed for reporting programme indicators to GF and PEPFAR. Government forms often did not allow for collection of data on peer‐led outreach, and data were often not disaggregated for KPs. Information was not routinely shared between implementing partners. This could lead to data duplication on individuals receiving services, making it difficult to generate reliable data on the performance of partners across the cascade 40. Clients sometimes used more than one identity or identifying feature, and this too could cause duplication of individuals in recording systems. The lack of unique identifier codes (UICs), or the existence of multiple UICs among different providers, also contributed to this problem.

Half of the assessments found that data were often not analysed routinely to guide programme implementation, due to high workloads and an inability to process data and generate usable insights from them. Data security was also a challenge, with data not consistently kept secure on computers or in hard copy, or not backed up.

Teams recommended coordination of data reporting systems through regular meetings of stakeholders at the national level, and technical working groups to design reporting systems to harmonize data collection needs, standardise tools, and reduce duplication of KP records. Policies and procedures on data security and confidentiality were also recommended. Adoption or expansion of UICs was discussed with all countries to ensure that individual KP members could be tracked across the cascade of services, and to avoid double‐counting individuals who received services at different times or locations, or from different funders 41.

### 2.6 Stigma, discrimination and violence

In all countries, programmes reported that KPs were subject to stigmatization and discrimination from multiple sources, including law enforcement, health care providers, family members, and members of the wider community, as well as within KP communities themselves. Members of any of these groups, plus clients of sex workers, were sometimes perpetrators of violence. Violence, stigma and discrimination deterred KP individuals from seeking services and increased their vulnerability to HIV and other sexually transmitted infections (STIs) 42, 43. KPs suffered from lack of police protection, weak enforcement of anti‐discrimination laws where these existed, and lack of awareness of their rights. Cases of violence frequently went unreported to the authorities or were not properly recorded, making it hard to advocate for resources or systemic efforts to reduce violence. There was also limited access to PEP for KP individuals who suffered sexual assault, often because its availability was not promoted or understood.

Recommendations for all countries included building on existing relationships with police and healthcare workers to offer sensitization on violence, stigma, discrimination and the rights of KPs to access HIV and other services. Work was also recommended with KP groups to better understand their rights as citizens, and to learn not to consider violence as normal or inevitable. Community‐based violence response systems using best practices were proposed 44, 45.

### 2.7 Community empowerment and engagement

The assessments recommended that drop‐in centres and other safe spaces should be established to provide KP communities a place to gather and to build a sense of community. The systematic engagement of community advisory boards in the KP programmes was another recommendation to strengthen a KP‐led response. POWs and navigators are key parts of the HIV programme, and in countries where there was high turnover, burnout, or poor motivation, it was recommended to boost training and supportive supervision, consider the selective use of incentives, and develop opportunities for progression to roles of increasing responsibility within CBOs and NGOs 8, 9, 10, 11, 12.

## 3 Summary

The joint cascade assessments offered countries candid feedback on the challenges of their HIV programmes and recommendations for ways to strengthen them at national and local levels. Areas highlighted included: (1) the need for robust population size estimates to inform targets and assign resources for outreach, such as an adequate number of POWs and other staff to provide necessary levels of prevention, testing and ART adherence support; (2) the usefulness of microplanning to provide accessible and KP‐friendly services most relevant to individuals and to increase retention in the prevention and clinical cascade; (3) the importance of decentralized, community‐based services for HIV testing and ART, and of introducing new approaches such as self‐testing and PrEP; (4) the need to address the structural issues of stigma, discrimination, and violence against KPs to create a more enabling environment; and (5) the need for more effective and continual tracking of KPs across the cascade of services, and for monitoring tools and reporting systems to be coordinated between donor‐funded and national programs.

## 4 Limitations

The assessments have several limitations, including the lack of generalizability both within and among countries and their focus on only some of the WHO‐defined KP groups. The cascade assessments are not generalizable within the country where they were conducted, since they are meant to be a quick stocktaking exercise of existing KP service delivery sites chosen by convenience sampling. Since the KP implementers selected sites where they were operating programmes, it may be that services there were more KP friendly than in areas where these implementers were not active, although in planning the assessments, both higher and lower performing sites were targeted. In addition, the cascade assessments were conducted predominantly in Africa, and their findings and conclusions are thus skewed to that geographical context where programmes may be more nascent than in other regions.

The assessments were conducted over a six‐ to ten‐day period, often with three days devoted to site visits outside of the capital city, and teams often covered multiple types of KP programmes. The combination of the short duration of the assessments and visiting multiple KP programmes resulted in higher level, rather than specific, observations. The assessments were not designed to provide combined cascade estimates but did review programme data and data systems to align data collection methods and plan for joint analyses. Donors and ministries of health often chose which KPs would be the focus of the assessments, which resulted in people who inject drugs being underrepresented and no prisoners being included. None of the programmes in Africa had dedicated programming for transgender populations, but following the assessments, several programmes introduced disaggregated monitoring of transgender populations separate from MSM data 11, 46. No follow‐up assessments in the countries have been conducted to date, which makes systematic review of progress challenging. Follow‐up assessments were recommended to continue to monitor and develop KP programs.

## 5 Conclusion

In recent years, international donors and national governments have allocated additional resources to KP programmes to address the higher level of HIV incidence and prevalence among KPs. Reducing HIV transmission and acquisition within these populations will increase the equity and impact of broader national efforts. To ensure the impact of KP programmes, it is important to strategically invest the limited resources available. The assessment teams and country stakeholders viewed the country KP cascade assessments as an innovative model to align GF and PEPFAR in countries with shared KP investments. They may be a best practice for coordinating donor‐funded programmes that may overlap or inefficiently serve KPs.

Joint assessments help initiate a consensus vision between the major donors on how to design and implement their national KP programmes. They also help garner government support for recommendations, and result in on‐the‐ground action when headquarters and country‐level staff from both donors and government conduct the assessments together. For example, in Cameroon, the PEPFAR‐ and GF‐funded partner signed a letter of collaboration to align geographic areas, service packages, training, monitoring tools and the use of UICs. Key recommendations from Nepal's joint cascade assessment were included in the country's National HIV Investment Plan 2016‐2021, with standardization of the cascade framework across funders. Swaziland's assessment helped the national programme understand KP programming as relevant to include within the entire country HIV cascade, and the recommendations were used to harmonize and expand the emerging GF programmes.

While agreement between global and national actors empowers implementers to put recommendations into action at the local level to develop a more efficient and technically sound KP programme, cascade assessments are just one step in the right direction. Global and national HIV programmes need investment of time, resources, and commitment from stakeholders to continually course‐correct, to align and improve programmes for sustained impact. The type of continued partnership demonstrated by these joint GF and PEPFAR assessments is key.

## Competing interests

The authors declare that they have no competing interests.

## Authors’ contributions

TL, DA and CW developed the concept for this article. TL and JB drafted and edited the manuscript. DA, JZ and CW reviewed and commented upon the manuscript at each stage of drafting.

## Funding

USAID, with support by PEPFAR, funded level of effort for Joint GF/PEPFAR KP HIV Cascade Assessments. The Global Fund supported the cascade assessments through staff time and logistical support (e.g. vehicles).

## Disclaimer

The views expressed in this paper do not necessarily reflect the views of the U.S. President's Emergency Plan for AIDS Relief, the U.S. Agency for International Development or the U.S. Government.
